# Harnessing Plant Sugar Metabolism for Glycoengineering

**DOI:** 10.3390/biology12121505

**Published:** 2023-12-08

**Authors:** Sophia N. Tang, Collin R. Barnum, Matthew J. Szarzanowicz, Sasilada Sirirungruang, Patrick M. Shih

**Affiliations:** 1 Department of Molecular and Cell Biology, University of California, Berkeley, CA 94720, USA; sophiatang@berkeley.edu; 2 Feedstocks Division, Joint BioEnergy Institute, Emeryville, CA 94608, USA; m_szarzanowicz@berkeley.edu (M.J.S.);; 3 Environmental Genomics and Systems Biology Division, Lawrence Berkeley National Laboratory, Berkeley, CA 94710, USA; 4 Biochemistry, Molecular, Cellular and Developmental Biology Graduate Group, University of California, Davis, CA 95616, USA; 5 Department of Plant and Microbial Biology, University of California, Berkeley, CA 94720, USA; 6 Innovative Genomics Institute, University of California, Berkeley, CA 94720, USA

**Keywords:** glycoengineering, metabolic engineering, glycoconjugates, glycosides, carbohydrates, nucleotide sugars

## Abstract

**Simple Summary:**

Sugars are one of the fundamental building blocks of life, but despite their essentiality, only a limited number of polysaccharides and glycoconjugates can be made synthetically. Plants use photosynthesis to produce a vast array of sugar-derived compounds in large quantities, while other means of production, such as chemical synthesis or microbial fermentation, are narrow in their range of sugar chemistries and comparatively low in yield. These qualities make plants an attractive platform for the synthesis of sugars and other glycosylated products. Plants have already been engineered to make products composed of or containing sugars that otherwise may be challenging to synthesize in other commonly used systems. Their growing use in glycoengineering efforts will continue to expand the production of diverse sugar-derived compounds.

**Abstract:**

Plants possess an innate ability to generate vast amounts of sugar and produce a range of sugar-derived compounds that can be utilized for applications in industry, health, and agriculture. Nucleotide sugars lie at the unique intersection of primary and specialized metabolism, enabling the biosynthesis of numerous molecules ranging from small glycosides to complex polysaccharides. Plants are tolerant to perturbations to their balance of nucleotide sugars, allowing for the overproduction of endogenous nucleotide sugars to push flux towards a particular product without necessitating the re-engineering of upstream pathways. Pathways to produce even non-native nucleotide sugars may be introduced to synthesize entirely novel products. Heterologously expressed glycosyltransferases capable of unique sugar chemistries can further widen the synthetic repertoire of a plant, and transporters can increase the amount of nucleotide sugars available to glycosyltransferases. In this opinion piece, we examine recent successes and potential future uses of engineered nucleotide sugar biosynthetic, transport, and utilization pathways to improve the production of target compounds. Additionally, we highlight current efforts to engineer glycosyltransferases. Ultimately, the robust nature of plant sugar biochemistry renders plants a powerful chassis for the production of target glycoconjugates and glycans.

## 1. Introduction

Glycosylation is a key step in the formation of numerous biologically and economically important compounds ranging from complex polysaccharides to diverse small molecules. Due to the difficulties involved in in vitro synthesis, major investments have been made to engineer biosynthetic pathways into industrially relevant species such as *E. coli* or *S. cerevisiae* to produce glycosylated compounds; however, plants are often overlooked as a production platform. Engineering within plants is still in its nascent stages relative to the microbial realm, but the potential for bioengineering in this field is tremendous, especially with regard to glycoengineering.

**Figure 1:**
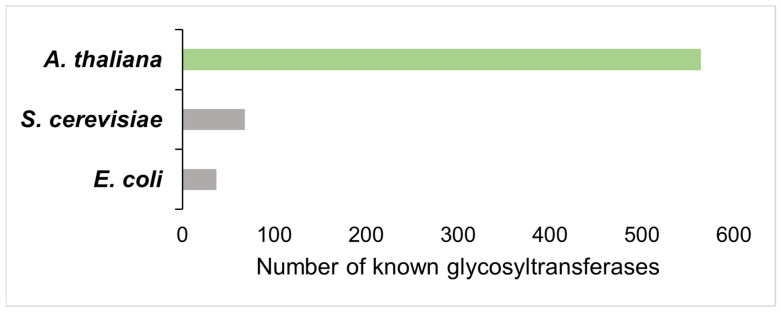
Plants encode dramatically more glycosyltransferases than other traditional model organisms used for metabolic engineering. Number of known glycosyltransferases in different model organisms. Data acquired from the Carbohydrate Active EnZYmes (CAZY) Database.

Plants have a natural propensity for sugar metabolism. As the predominant global primary producers of our planet, plants derive the vast majority of their biomass from precursor sugars produced through photosynthesis, requiring robust sugar biosynthesis, transport, and manipulation. The scale and productivity of plant sugar metabolism are most readily demonstrated by the fact that plants constitute 80% of all biomass on Earth [1], most of which is captured in the form of polysaccharides. Plants’ various carbohydrates and glycoconjugates are biosynthesized by glycosyltransferases (GTs), a large group of enzymes that has dramatically diversified in land plants. For comparison, the model plant *Arabidopsis thaliana* encodes an estimated 564 glycosyltransferases, whereas the model microbes *Escherichia coli* and *Saccharomyces cerevisiae* possess only 37 and 66 GTs, respectively, demonstrating a vast disparity in the extensiveness of potential endogenous glycosylation (Figure 1).

Although plants are notable for their polysaccharides, this opinion piece will largely focus on plants as production platforms for glycoconjugates, as polysaccharide engineering for a variety of purposes, including biofuels production, carbon sequestration, and human health, has been reviewed extensively elsewhere [2,3,4,5,6]. The literature compiled herein demonstrates that plants are uniquely positioned for the photosynthetically driven production of a wide diversity of glycoconjugates and glycans as an extension of their already robust sugar metabolism.

## 2. Engineering Nucleotide Sugar Biosynthesis

**Figure 2:**
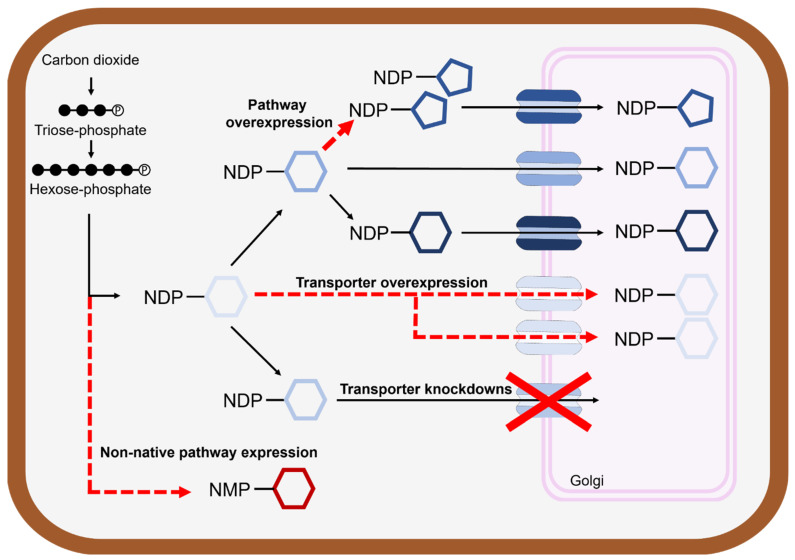
Strategies for nucleotide sugar engineering. Nucleotide sugars serve as a critical input for the production of many important biomolecules. Pathway overexpression, transporter overexpression, transporter knockdowns, and the inclusion of non-native nucleotide sugar biosynthesis pathways are approaches that can be used to improve nucleotide sugar availability for product synthesis.

While glycosylated biomolecules have diverse structures and functions, their sugar moieties are derived from the same core set of nucleotide sugar precursors. Nucleotide sugars are activated sugars consisting of a nucleoside monophosphate (NMP) or nucleoside diphosphate (NDP) and a monosaccharide. As the primary sugar donor for the production of glycosylated biomolecules, nucleotide sugars act as an interface between primary and specialized metabolism. The biosynthesis of these activated sugars begins from photosynthetically derived triose/hexose phosphates that are then fed into a variety of branched pathways that attach a single sugar moiety to a respective nucleotide. Additionally, free sugars in the plant can be recycled into the nucleotide pool via salvage pathways. There are also several key nucleotide sugars that can be interconverted into another, serving as branch points in the synthesis of other nucleotide sugars (Figure 2) (See [7,8] for in-depth reviews on plant nucleotide sugar metabolism). These branches in the pathway invite questions about how the concentration of an upstream precursor nucleotide sugar can influence the levels of downstream nucleotide sugars. Limited studies have investigated this question; however, some engineering efforts have demonstrated surprising plasticity in plant nucleotide sugar biosynthesis. The overexpression of two *Arabidopsis thaliana* genes, a UDP-glucose 4-epimerase used in the conversion of UDP-glucose to UDP-galactose and a β-1,4-galactan synthase, increased cell wall galactose monosaccharide composition by 80% without significantly altering levels of other UDP-glucose-derived monosaccharides [9]. While additional studies are needed, this suggests that plants are able to accommodate metabolic alterations to native nucleotide sugar levels without major effects on other important end-product biomolecules.

**Figure 3:**
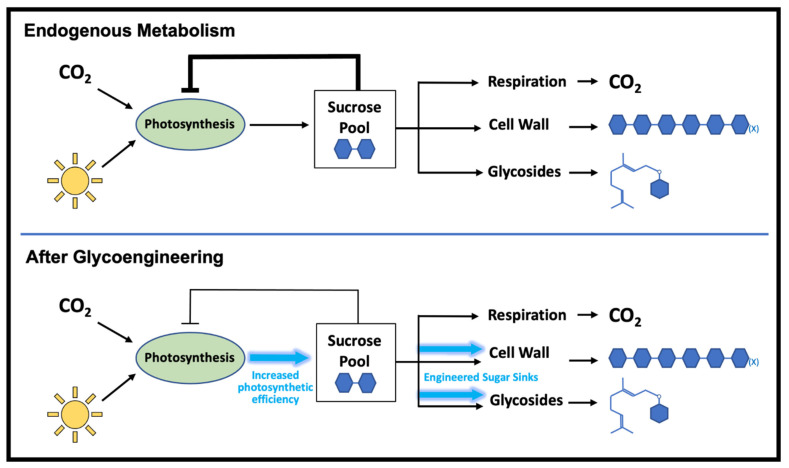
Autoregulation of plant source–sink relationships. Sucrose serves as the general carbon precursor to all plant growth and development, and an accumulation of sucrose has an inhibitory effect on the photosynthetic efficiency of a plant. Creating additional nucleotide sugar sinks that deplete cellular sucrose levels stimulates photosynthesis and increases plant sucrose production. This innate compensatory effect allows for the engineering of additional sugar sinks without the depletion of precursor sugar substrates.

In addition to being able to tolerate substantial changes in sugar biosynthesis, the creation of new nucleotide sugar sinks can actually improve total sugar production. Although cellulose is already the greatest carbon sink in plants, comprising 40–50% of plant biomass, plants are able to tolerate additional sugar allocation to cellulose biosynthesis [10]. The heterologous overexpression of a cellulose synthase gene from *Ciona savignyi* (sea squirt) in sugarcane increased cellulose content by up to 31% and 28% in young and mature internodes, respectively [11]. Interestingly, the increased use of UDP-glucose for cellulose production did not decrease sucrose despite sucrose being the precursor to nucleotide sugars. Rather, sucrose and other free sugar levels in fact increased with the stronger sink, suggesting that plants have the capacity to respond to perturbations in nucleotide sugar usage by stimulating increased sucrose production to restore nucleotide sugar levels (Figure 3).

Such a phenomenon has been observed in other species through the overexpression of sucrose synthase genes that catalyze the reaction of sucrose and UDP to form UDP-glucose and fructose. Expressing *SUS5* from bamboo in poplar led to an increased growth rate and cellulose content—presumably from increased UDP-glucose levels—along with a concomitant increase in photosynthetic rate to adapt to the stronger carbon sink [12]. Similar increases in biomass accumulation and photosynthetic rate following the overexpression of sucrose synthases were seen in tobacco [13,14], and the relationship between sucrose utilization and increased photosynthetic efficiency has been studied in sugarcane [15].

These lines of evidence suggest that plants are able to compensate for the metabolic burden of increased carbon channeling to nucleotide sugars through increases in photosynthetic flux. This allows plants to metabolically accommodate sugar engineering efforts and sugar flux perturbations without the need to re-engineer upstream carbon fixation pathways, making plants an amenable chassis for sugar bioengineering. Such plasticity to sugar perturbations could allow for multiple nucleotide sugar biosynthetic pathways to be overexpressed simultaneously, thereby drawing on the abundance of carbon in plants to increase available nucleotide sugars for product formation. This approach could be coupled with strategies to increase the availability of nucleotide sugar precursors, such as the overexpression of invertases, to optimize carbon flux to a product of interest.

## 3. De Novo Nucleotide Sugar Engineering

In addition to the optimization of canonical nucleotide sugar production, plants can be engineered to make non-native nucleotide sugars (Figure 2). Cytidine-5′-monophospho-*N*-acetylneuraminic acid (CMP-Neu5Ac) is an activated sialic acid present in mammals that is required for the biosynthesis of glycosylated mammalian proteins. It is now believed that CMP-Neu5ac is not natively created in plants [16], which has hindered the use of plants as a production platform for mammalian proteins. Castilho et al. [17] utilized transient expressions in *Nicotiana benthamiana* to reconstitute the mammalian CMP-Neu5Ac pathway *in planta*. Through the expression of six mammalian enzymes that localized to the nucleus, cytosol, or Golgi, CMP-Neu5Ac was synthesized, transported into the Golgi, and used to glycosylate a functional human monoclonal antibody [17]. This enables the production of sialylated small molecules and proteins, thereby expanding the role of plants as a chassis for the production of commodity biomolecules. These efforts expand the metabolic capacity of plants through the introduction and use of heterologous nucleotide sugar pathways.

## 4. Engineering Nucleotide Sugar Transport and Localization

While nucleotide sugar biosynthesis largely occurs in the cytosol, the Golgi and endoplasmic reticulum (ER) are major sites of glycosylation. In many cases, nucleotide sugar transporters (NSTs) are required for the transportation of these compounds across the membranes of the Golgi and ER for the production of glycans and certain nucleotide sugars, such as UDP-galacturonic acid and UDP-arabinopyranose [18]. The discovery of many new NSTs in recent years [19,20,21] has enabled their use in pathway engineering to alter the availability of nucleotide sugars in different cellular compartments (Figure 2).

NST overexpression and knockdowns could be used to enhance the availability of nucleotide sugars in the Golgi and in the cytoplasm, respectively, to increase substrate concentrations for synthesizing cell wall components or glycoconjugates. The overexpression of UDP-arabinose transporters, UAfT2, UAftT3, or UAft4, in *Arabidopsis thaliana* increased arabinose concentration in cell wall extract by up to 30% [20]. Conversely, NSTs could be knocked down to increase cytosolic levels of particular nucleotide sugars. However, NST manipulation could result in phenotypic abnormalities [20] or limit plant fitness [21]. Alternative strategies to optimize production across organelle boundaries could include relocalizing cytosolic pathways that require nucleotide sugars to the Golgi or ER, circumventing the need to knockdown NSTs.

## 5. Engineering Plant Glycosyltransferases

GTs utilize nucleotide sugar precursors to decorate proteins, glycans, and specialized metabolites with a monosaccharide. While there are diverse families of glycosyltransferases, GT super family 1 is of particular interest when examining specialized metabolism. GTs from super family 1 are termed UDP-dependent glycosyltransferases (UGTs) as they glycosylate small molecules using UDP-sugars. UGTs have been found to facilitate the formation of O-, N-, S-, and C-glycosides of a large repertoire of sugar acceptor substrates, including flavonoids, alkaloids, terpenoids, polyphenols, glycosides, as well as synthetic compounds. Notably, UGTs have been discovered that are active on industrially relevant molecules such as the indigo precursor indoxyl [22], cannabidiol [23,24], and the non-caloric sweetener precursor steviol [25]. In fact, a recent screening effort demonstrated an unprecedented promiscuity of many UGTs, suggesting that a much broader scope of molecules can be enzymatically glycosylated than previously believed [26]. This promiscuity can enable advances in the use of UGTs in biotechnology. As glycosides of small molecules often possess physical and biological properties distinct from their aglycone counterparts, UGTs provide a powerful tool to alter a metabolite’s localization, transport, storage, and activity.

In addition to discovering additional UGTs, engineering efforts have generated UGTs with novel functions. Such advances have been assisted by structural information, which provides a basic understanding of UGT chemoselectivity [27,28,29], regioselectivity [30,31,32], sugar donor selectivity [33], and sugar acceptor selectivity [34,35]. For example, Wetterhorn et al. engineered UGT Os79 from rice, which natively glycosylates a disease-causing mycotoxin, deoxynivalenol, to accommodate a bulkier analog, T-2 toxin, paving the way to protect crops against a broader scope of fungal infections [36]. He et al. demonstrated a shift from C- to O-glycosylation activity of TcCGT1 on substrate flavones apigenin and luteolin [28]. Li et al. were able to achieve a near-perfect regiocontrol of glycosylation of silybin A via rational engineering of UGT74AC2, which natively produces a mixture of products [37]. Notably, UGTs involved in C-glycosylation, which commands much attention due to the creation of stably bound glycoside products, have been more recently characterized in depth [38,39,40]. Although less than a hundred related CGT enzymes have been discovered, the number of molecules that can be enzymatically C-glycosylated and our understanding of their mechanism are steadily increasing [41]. Such advances in plant GT engineering demonstrate tremendous diversity and plasticity in UGTs, suggesting an immense opportunity in the design and assembly of metabolic pathways that utilize the vast nucleotide sugar pools of plants.

In addition to GTs, glycosyl hydrolases (GHs), which typically catalyze the hydrolysis of glycosides and glycans, have been shown to possess biosynthetic capabilities, further expanding ways to engineer sugar metabolism in plants [42,43].

## 6. Glycoconjugate Biosynthetic Pathway Engineering

Plants natively contain hundreds of GTs, raising the question of if and how metabolic crosstalk may hinder engineering efforts through off-target reactions and final yield depletion. An attempt to synthesize the artemisinin precursor artemisinic acid in *N. benthamiana* resulted in product glycosylation to artemisinic acid-1,2-β-diglucoside, demonstrating the effects of promiscuous native GTs on foreign metabolites [44]. Similarly, the undesired effects of native glycosyltransferase activity were seen in *N. benthamiana* used to produce octaketide anthraquinones, suggesting that such crosstalk glycosylation events may impact a diverse range of metabolic products [45]. However, through the additional expression of a foreign glycosyltransferase, *DcUGT2*, Andersen-Ranberg et al. [45] were able to channel flux toward the anthraquinone of interest, demonstrating the capacity of highly expressed transgenes to compete with native host metabolism. The persistence of some undesired glycosylation events produced from endogenous GTs implies that further refinements can be made through targeted knockouts to channel flux to engineered pathways of interest.

Examples of such knockouts are well defined in the field of plant glycoprotein engineering. A recent study produced recombinant human proteins devoid of α-1,3-fucose and β-1,2-xylose linkages in *N. benthamiana* through knockouts of six endogenous GTs [46]. Human-like glycosylation has also been achieved in *Physcomitrella patens* by knocking out a α-1,4-fucosyltransferase and a β-1,3-galactosyltransferase while expressing a human β-1,4-galactosyltransferase [47]. Such examples demonstrate that plant sugar metabolism can accommodate engineered perturbations via GT overexpression and knockouts, making plants a versatile chassis for glycosylated biomolecule productions (reviewed by Margolin et al. [48]).

## 7. Conclusions

Plant glycoengineering presents many opportunities to redirect photosynthate towards a broad range of bioproducts. Plants innately possess a tremendous capacity to produce and manipulate sugars, and thus, they present a promising platform for glycoengineering. The metabolic capacity to tolerate and balance nucleotide sugar flux perturbations without the need to re-engineer upstream metabolism, including photosynthesis, is of particular note and suggests that plant metabolism is amenable and robust in the face of genetic manipulation. Additionally, the plant tolerance of GT knockouts and heterologous expression further demonstrates that plants are a versatile chassis for glycosylated biomolecule production.

While many advances have been made, few have unified the aforementioned techniques into a stably transformed system. Future work by the glycoengineering community will require an integration of the engineering strategies mentioned here, including channeling flux through nucleotide sugars, creating knockout lines of competing pathways, and integrating tailored GTs stably into the genome to fully realize the potential of plants as platforms for metabolic glycoengineering.

## Data Availability

Not applicable.
